# Human mobility in large cities as a proxy for crime

**DOI:** 10.1371/journal.pone.0171609

**Published:** 2017-02-03

**Authors:** Carlos Caminha, Vasco Furtado, Tarcisio H. C. Pequeno, Caio Ponte, Hygor P. M. Melo, Erneson A. Oliveira, José S. Andrade

**Affiliations:** 1 Programa de Pós Graduação em Informática Aplicada, Universidade de Fortaleza, Fortaleza, Ceará, Brasil; 2 Departamento de Ensino, Instituto Federal de Educação, Ciência e Tecnologia do Ceará, Crateús, Ceará, Brasil; 3 Departamento de Física, Universidade Federal do Ceará, Fortaleza, Ceará, Brasil; Northwestern University, UNITED STATES

## Abstract

We investigate at the subscale of the neighborhoods of a highly populated city the incidence of property crimes in terms of both the resident and the floating population. Our results show that a relevant allometric relation could only be observed between property crimes and floating population. More precisely, the evidence of a superlinear behavior indicates that a disproportional number of property crimes occurs in regions where an increased flow of people takes place in the city. For comparison, we also found that the number of crimes of peace disturbance only correlates well, and in a superlinear fashion too, with the resident population. Our study raises the interesting possibility that the superlinearity observed in previous studies [Bettencourt *et al*., Proc. Natl. Acad. Sci. USA **104**, 7301 (2007) and Melo *et al*., Sci. Rep. **4**, 6239 (2014)] for homicides versus population at the city scale could have its origin in the fact that the floating population, and not the resident one, should be taken as the relevant variable determining the intrinsic microdynamical behavior of the system.

## Introduction

The dynamics of crime and the impact of social relations on the increase of violence has been the object of study in several areas such as Social Sciences [1], Criminology [2–4], Computing [5–9], Economics [10] and Physics [11–17]. When, in the 1950s, Naroll and Bertalanffy [18] utilized the concept of allometry—a term originally coined in the field of Biology to describe scaling laws, *e.g*., the relationship between mass and metabolic rate of organisms [19]—so as to adapt it to the social context, a particularly promising line of research was opened, which today arouses interest of scientists from wide-ranging areas [11, 20–32]. In 2007, Bettencourt *et al*. [21] revealed that allometric relationships are statistically present in many aspects of city infrastructures and dynamics. These allometric relationships could be described by a power law function, *Y* = *aX*^*β*^, where usually *X* is the population, *Y* is a social indicator, *a* is a constant and *β* is the allometric exponent. In particular, they observed a characteristic superlinear relation between the number of serious crimes and the resident population in the United States (US) cities which clearly denotes the an intricate social mechanism behind the dynamics of violence. Recently, Bettencourt *et al*. [22] and Melo *et al*. [11] provided strong quantitative evidence showing that an similar behavior can also be observed in Brazil.

Despite the importance of the results presented in the aforementioned studies, their impacts on urban planning, more specifically, on the development of public safety policies, are limited due to its purely descriptive nature, which prevents a deeper understanding of the organic causes leading to such a disproportional behavior. In this way, a microdynamical approach based on the interactions between local neighborhoods within metropolitan areas certainly represents a more realist view to the problem. Our research motivation is aimed at elucidating issues related to the understanding of the impacts of the influence of social relations on Crime. In Criminology, the role of urban space and its social relations has been previously emphasized to explain the origin of Crime [2, 4]. Particularly, the routine activity theory, proposed by Cohen and Felson [2], states that crimes, more specifically property crimes, such as robbery and theft, occur by the convergence of the routines of an offender, motivated to commit a crime, and an unprotected victim. In this context, can we explain the occurrence of crimes in different areas of the city based on the current population present in the corresponding urban sub-clusters? Is this effective population equally important for any type of crime? How can we systematically delimit the boundaries of these local neighborhoods so that the social influence—the inherent correlations among social indicators—be accounted for in a consistent way?

In order to answer these questions, we used actual georeferenced data of crimes committed, and of resident and floating populations for census tracts in the city of Fortaleza, Brazil. The concept of resident population has been widely used to understand the effects that the growth of major cities has on social and environmental indicators. In particular, Bettencourt *et al*. [21] showed that the number of homicides scales superlinearly with the population of cities in the US. Subsequently, Melo *et al*. [11] confirmed this behaviour for Brazilian cities, but also demonstrated that suicides scale sublinearly with their resident populations. Additionally, Oliveira *et al*. [20] found a superlinear allometric relationship between the resident population and CO_2_ emissions; this study also raised the issue that the allometric exponents may undergo endogenous trends depending on the respective definition of urban agglomerates. Regarding the floating population, it takes into account the complex dynamics of urban mobility, *i.e*., it has a transient characteristic within the city. To measure social influence quantitatively in urban sub-clusters, we delimited their boundaries beyond the mere administrative divisions, *e.g*. division by neighborhoods or census tracts, by using the City Clustering Algorithm [33–40]. This model defines these boundaries by population density and the level of commuting between areas of the city.

In the present study, we identified that the incidence of property crimes has a superlinear allometric relationship, with the floating population in certain areas of the city. This result implies that the increased flow of people in a particular area of the city will take place at the cost of a proportionally greater rate of property crime happening in the region. More important, this superlinear behavior at the subscale of the city neighborhood provides a plausible explanation for the allometry of serious crimes found in [11, 21]. Precisely, the floating population being systematically larger that the resident one should lead to the disproportional behavior observed for serious crimes and (resident) population at the city scale. We also found a superlinear allometric relationship between the number of crimes of disturbing the peace and the resident population. This result shows that the effect of social influence must be adequately correlated with resident or floating population, depending on the type of crime considered.

## Datasets

In order to quantify the effects of the social influence on the police calls within a large metropolis, we used three georeferenced datasets for the Brazilian city of Fortaleza: From the first, we obtain the *resident population* (POP), defined by the number of residents per *census tract*—Administrative territorial unit established for the purposes of cadastral control—and provided by the Brazilian Institute of Geography and Statistics (IBGE) [41]. In all, Fortaleza has 3043 census tracts, with approximately 2,400,000 residents, spread over an area of 314 square kilometers (km^2^) in year 2010. From the second, we estimate the *floating population* (FLO) for each census tract through a flow network built on data of the bus system provided by the Fortaleza’s city hall for the year 2015 [42]. FLO was measured by the number of people who pass through a census tract in one day. The city of Fortaleza has 2034 buses circulating along 359 bus lines serving approximately 700,000 people who use the city’s mass transit system on a daily basis. In the case of Fortaleza, buses still represent the main means of public transportation. The process of generating of the flow network will be detailed in the Supporting Information (see S1 Appendix). Finally, we obtain the *Crime dataset* from the Integrated Coordination Office of Public Safety Operations (CIOPS) [43], provides the geographic locations of 81,911 calls to the “190” (phone number for emergency) service about property crimes (PC) and 53,849 calls to the same service about disturbing the peace (DP). These calls were made to police between August 2005 and July 2007. Here we assume that massive changes in the urban environment of the city have not been produced in the period under investigation (5 to 8 years). This would not be that case if the society were facing a severe social rupture due, for example, to natural disasters, economic embargoes or wars. See S1 Appendix for information about all datasets utilized in this study, including the URL for downloading.

**Fig 1:**
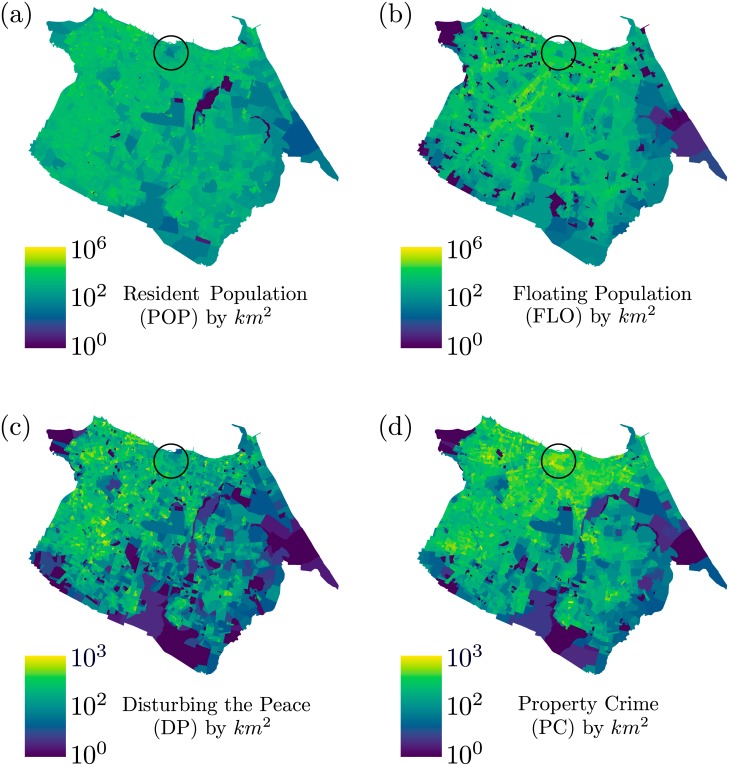
The density maps for the city of Fortaleza (Brazil) in logarithmic scale. (a) The resident population (POP) by square kilometers (km^2^). (b) The floating population (FLO) by km^2^. (c) The disturbing the peace (DP) complaints by km^2^. (d) The property crimes (PC) by km^2^. The black circle highlights the downtown area of the city. This region has a low density of residents and disturbing the peace calls, and is dense in the flow of people and property crimes.

The color maps show in Fig 1(a)–1(d) provide the local density in logarithmic scale of POP, FLO, DP and PC, respectively, for the city of Fortaleza. We can clearly identify stronger spatial correlations between POP and DP as well as between FLO and PC. In special, there is an evident higher incidence of hot spots in (b) and (d) than in (a) and (c). Also, at the downtown area, highlighted by black circles on each map, the high density of FLO is compatible with the high rates of PC rate, while low POP densities seem to explain the low frequency of DP complaints.

**Fig 2:**
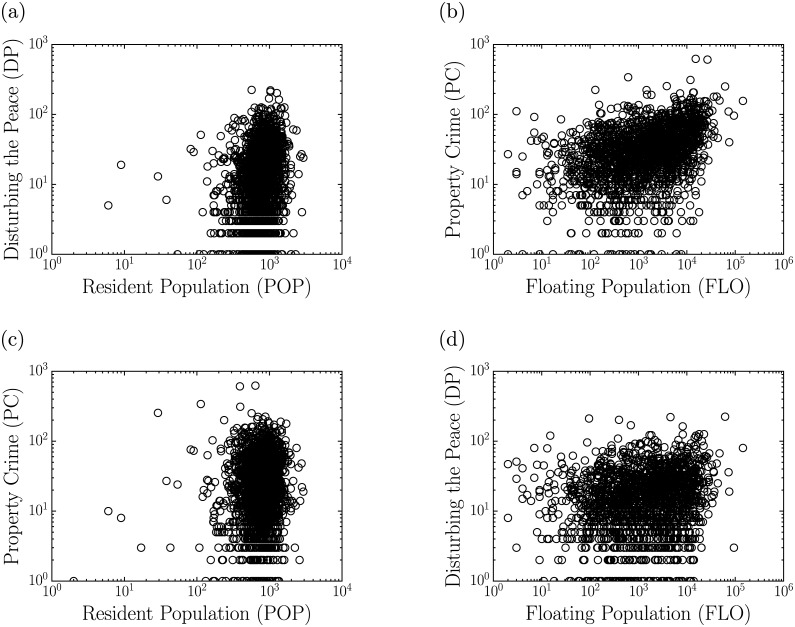
Scattering plots by census tracts. (a)-(d) All the plots between resident population (POP) and floating population (FLO) with police calls for disturbing the peace (DP) and for property crimes (PC) show uncorrelated behavior with the determination coefficients [44, 45] *R*^2^ < 0.15.

In spite of seeming trivial to suggest that there are correlations between POP and DP, as well as FLO and PC from the density maps shown in Fig 1, the respective scattering plots of the Fig 2 fail to capture the spatial correlations presented in Fig 1 due to the non-aggregation of the datasets of both populations. Actually, we conjecture that such correlations exist indeed. The most census tracts have a small area, sometimes the size of one city block, and it is likely that such an agglomeration scale is insufficient to capture the correlations and therefore reveal the impact of social influence on DP and PC, additionally the population tends to be equally distributed among its census tracks and this probably also has influence over the weak correlation presented in the Fig 2. Based on this hypothesis, we considered coarse-grainning these spatial properties of Fortaleza into clusters, using the census tracts grid as a maximal resolution base. This clustering aims to find the boundaries of the flow of people and the residential areas of the city.

## Methods

**Fig 3:**
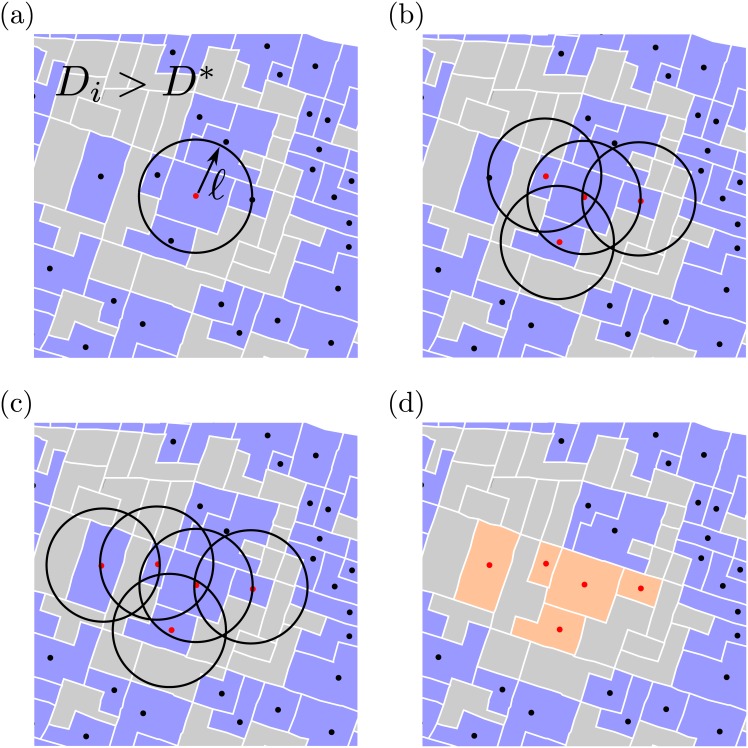
The scheme of the City Clustering Algorithm (CCA). Each polygon represents a clustering unit, specifically in our case, they represent census tracts. The light blue polygons are candidates for clustering (*D*_*i*_ > D*); in contrast, the gray polygons cannot be clustered (*D*_*i*_ ≤ D*). (a) The red dot represents the geometric center of the *i*–th census tract and the black circle with radius *ℓ* seeks neighbors belonging to the same cluster. (b)-(c) The same search operation is made for the other census tracts and is done until there are no more neighbors within the radius of operation. (d) The algorithm finishes running and the cluster is found.

To define the boundaries beyond administrative delineations, we considered the notion of spatial continuity through the aggregation of census tracts that are near one another using the City Clustering Algorithm (CCA) [33–40]. The CCA constructs the population boundaries of an urban area considering two parameters, namely, a population density threshold, D*, and a distance threshold, *ℓ*. For the *i*–th census tract, the population density *D*_*i*_ is located in its geometric center; if *D*_*i*_ > D*, then the *i*–th census tract is considered populated. The length *ℓ* represents a cutoff distance between census tracts to consider them as spatially contiguous, *i.e*., all of the nearest neighboring census tracts that are at distances smaller than *ℓ* are clustered. Hence, a cluster made by the CCA is defined by populated areas within a distance less than *ℓ*, as seen schematically in Fig 3. Previous studies [20, 37, 39] have demonstrated that the results produced by the CCA can be weakly dependent on D* and *ℓ* for some range of parameter values. Here *ℓ* will be quantified in meters (m) and D* in inhabitants by km^2^.

Although the algorithm begins collating an arbitrary seed census tract, it does not produce distinct clusters when varying this seed; the two factors that are responsible for clustering behavior are the parameters *ℓ* and D*.

**Fig 4:**
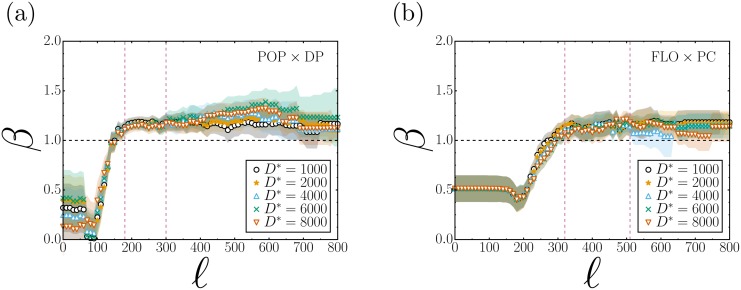
Behavior of exponent *β* by varying the parameters of the City Clustering Algorithm (CCA), *ℓ* and D*. (a) The variation of *β* in correlation between the resident population (POP) and the disturbing the peace (DP) complaints is illustrated; (b) The variation is illustrated for correlations between the floating population (FLO) and the property crimes (PC). Both in (a) and (b), the x-axis represents *ℓ*, and this parameter was varied from 0 to 800 meters (m) (moment when the largest cluster consumes nearly the entire city); exponent *β* is shown on the y-axis. The colors of the lines represent the variation of the parameter D*, which corresponds to the resident population density in (a) and the floating population density in (b); this parameter was varied from 1000 to 8000. The shadows represent the standard error of coefficient *β*. It was not necessary to use values larger than 8000 because many census tracts start being discarded and the CCA can no longer form clusters. The graphs also show red dashed lines; between these lines is highlighted the range where, regardless of the parameterization, the exponent *β* has smaller ranges of variation. Finally, the dotted black line highlights exponent *β* = 1, in which the relationship between variables is isometric, in both graphs the exponent oscillates to low values of *ℓ*; in (a), the relationship is superlinear starting at *ℓ* ≥ 180 m; however in (b), superlinearity appears at *ℓ* ≥ 320 m (see S1 Appendix for the plots POP vs PC and FLO vs DP).

In order to determine the effect of the parameterization on the value of the exponent *β*, we sought a range within the parameters where *β* has low sensitivity to this variation. Fig 4 shows the behavior of exponent *β* in function of the variation of the CCA parameters. This result could be compared with the variation of the allometric exponent for different process of urban agglomeration [27, 29]. As shown in Fig 4a and 4b, the value of the parameter *β* obtained from the least-square regressions to the data of POP against DP remains practically insensitive to the CCA parameters in the range 180 ≥ *ℓ* ≥ 300, regardless of the values of D* adopted in the estimation process. Moreover, the resulting average *β* = 1.17 ± 0.06 provides strong evidence to support a superlinear type of relation between these two variables. An entirely similar behavior can be observed for FLO against DP, but now the exponent *β* remains practically invariant within the range 320 ≥ *ℓ* ≥ 510. The resulting average value of *β* = 1.14 ± 0.04 also indicates the presence of a superlinear allometric relation.

At this point, it is necessary to determine a suitable criteria for selecting an adequate value of the parameter D*. While lower values of *ℓ* lead to the formation of a larger number of CCA clusters, reduced values of D* tend to eliminate fewer census tracts from the map, thereby including a larger portion of in the population under analysis. Here we propose that a proper choice of D* would be associated with a more homogeneous spatial distribution of the population [20]. More precisely, we seek for CCA clusters whose areas should scale as close to isometrically as possible with the population data, namely, as *Y* = *aX*^*α*^, with *α* ≈ 1, where *X* is the population, *Y* is the area (ARE) of the clusters km^2^, and *a* is constant.

**Fig 5:**
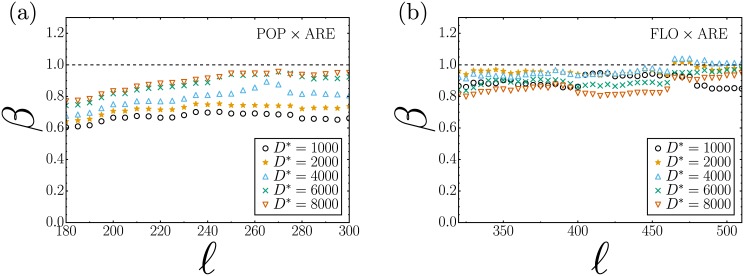
Behavior of exponent *α* when varying the City Clustering Algorithm (CCA) parameters *ℓ* and D*. (a) The variation of *α* in correlations between the resident population (POP) and the area (ARE) in square kilometers (km^2^) of clusters discovered with the CCA. (b) The variation for correlations between the floating population (FLO) and ARE. In (a) and (b), the x-axis represents the parameter *ℓ*, and the y-axis represents the exponent *α*. The line colors represent the variation of the parameter D*.

In order to follow the procedure previously described, we obtain from Fig 5a that *ℓ* = 270 and D* = 6000 correspond to the pair of CCA parameter values leading to the closest to isometric relation found between ARE and POP. In the case of ARE and FLO, the values are *ℓ* = 320 and D* = 2000, as depicted in Fig 5b (see S1 Appendix).

## Results

**Fig 6:**
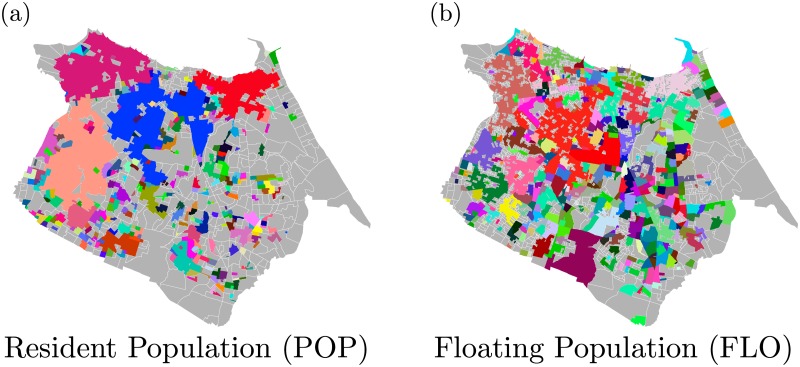
The City Clustering Algorithm (CCA) applied to resident population (POP) and to floating population (FLO). Each color represents a cluster; the light gray areas correspond to census tracts that were not grouped because they have *D*_*i*_ < D*. (a) The population density was used in order to find the boundaries of the clusters with *ℓ* = 270 m and D* = 6000 resident people per km^2^. (b) The division found by considering urban mobility is shown; the map illustrated here was generated for *ℓ* = 320 m and D* = 2000 floating people per km^2^ in one day in Fortaleza.

The census tracts were grouped, using the CCA, by POP and FLO (Fig 6). In the Fig 6a, the division achieved by POP is illustrated; the city was divided using *ℓ* = 270 m and D* = 6000 resident people per km^2^. In the Fig 6b, we show the division achieved by FLO, using *ℓ* = 320 m and D* = 2000 floating people per km^2^ per day. We emphasize that there are bigger gaps in the POP map (Fig 6a) than in the FLO map (Fig 6b). The reason for such behavior is the fact that Fortaleza has commercial regions, *i.e*., regions where there is not a large presence of residents. Regarding floating people, there are people moving practically throughout the entire city, both in commercial areas and in residential areas.

**Fig 7:**
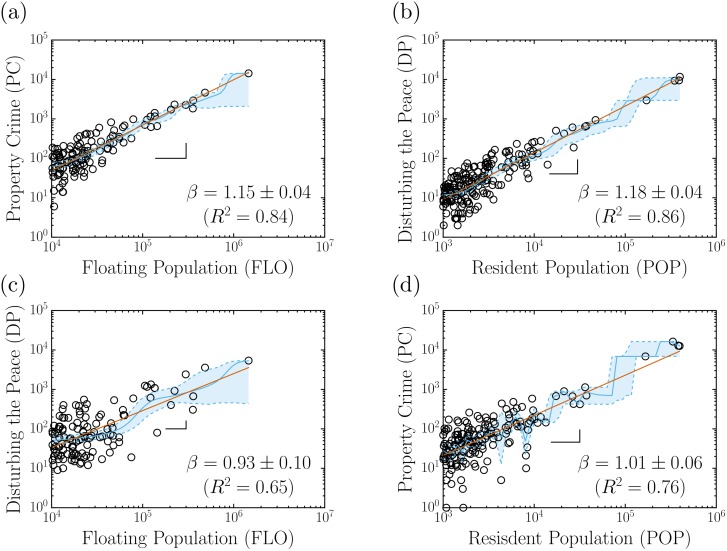
Scattering plots by City Clustering Algorithm (CCA). The red lines represent the simple linear regressions applied to the data, the blue continuous lines represent the Nadaraya-Watson method [46, 47] and the blue dashed lines delimit the 95% confidence interval (CI) estimated by bootstrap. (a) A superlinear relationship was found, with exponent *β* = 1.15 ± 0.04, between the floating population (FLO) and the property crimes (PC). (b) A superlinear relationship was also found between the resident population (POP) and the disturbing the peace (DP) complaints, with exponent *β* = 1.18 ± 0.04. (c)-(d) The scattering plots of DP with FLO and PC with POP show an isometric relation was found between the variables, but with lower correlations than (a) and (b). The *R*^2^ is defined as determination coefficient [44, 45].

As compared to the results shown in Fig 2, the application of the CCA to the data discloses a rather different scenario for the correlations among the variables investigated here. First, as shown in Fig 7a and 7b, superlinear relations in terms of power laws, *Y* = *aX*^*β*^, are revealed between PC and FLO as well as between DP and POP, with exponents *β* = 1.15 ± 0.04 and *β* = 1.18 ± 0.04, respectively. In contrast, the relations obtained between DP and FLO and PC and POP are closer to isometric (linear), with exponents *β* = 0.93 ± 0.10 and *β* = 1.01 ± 0.06 respectively, although the low values of the corresponding determination coefficients indicate that these results should be interpreted with caution.

## Conclusion

In this paper, we found allometric laws within a city using the CCA technique [33–40]. to determine intracity clusters. The volume of social influence was measured in various localities of the city based on the presence of residents and the flow of passers-by through the census tracts. Unlike intercity studies, where social influence was measured only by the presence of residents, we propose that, within a city, human mobility should be considered to understand the dynamics of social indicators, such as criminal activity. Our results show that the incidence of property crimes grows superlinearly as a power law with the floating population, with allometric exponent *β* = 1.15 ± 0.04. Therefore, the increase of the flow of people in a region of the city leads to a disproportionally higher number of this type of crime. Our results are in agreement with the routine activities hypothesis [2], which states that a crime occurs by the convergence of the routines of a motivated offender and an unprotected victim, as well as the absence of a guardian able to prevent the transgression. Apart from providing quantitative support for such hypothesis, namely, that the convergence of the routines of all agents increases the number of crimes, our study also indicates the remarkable fact that this increase is non-linear (*β* > 1). This result is in clear contrast with the incidence of crimes related with peace disturbance, where an allometric relation can also be detected, but with the resident population (POP) instead. We hope the our results could shed some light on the understanding of crimes inside urban areas, as well as assist eventual violence mitigation policies.

The findings described in this paper bring alternatives to implementing innovative practices to decision makers within cities. The most obvious of these relates to the fact that, by showing the correlation of different types of crimes with the home population but also with the floating population, it is also clear that the police force allocation strategies should be implemented via the analysis of different cluster configurations that depend on the type of crime. For example, the allocation of community policing, more appropriate to resolve conflicts that potentially can emerge from the disturbance of people’s peace, must be planned from a cluster configuration and a hot spot analysis that were produced from the perspective of the density of resident population. When it is necessary to establish a policy for allocating a uniformed police in order to mitigate crimes against property, the allocation of the police force must be conducted from an analysis from the movement of people.

In addition to these police allocation strategies, the results described herein provide important indicators for the creation of public policies for land use and environmental design in general. Work in this line has been developed such as in Ref. [48] where a framework has been proposed to associate the physical spaces and the feeling of safety as well as [49], who launched the environmental criminology putting focus of criminological study on environmental or context factors that can influence criminal activity. These include space (geography), time, law, offender, and target or victim. These five components are a necessary and sufficient condition, for without one, the other four, even together, will not constitute a criminal incident. The discovery demonstrated in the article that there is a superlinear relationship between crime and population (resident or floating) in clusters within cities strengthens the claim that changes in urban form can lead to reduced crime as discussed in Ref. [50]. In this context, we believe that there are two possible strategies to reduce crimes in the cities. In a short period of time, one could try to modify the route of public transportation in order to avoid a high spatial-temporal convergence of people. In a long period of time, by stimulating with tax breaks, for example, the existence of of several autonomous centers in the city, this would certainly reduce the commuting among these highly convergent centers.

## Supporting information

S1 Appendix The urban mobility data processing, lack of correlation between POP vs PC and FLO vs DP, additional information about the choosing of *ℓ* and D* and data summary. (PDF)
