# Supplementary material for: Human mobility in large cities as a proxy for crime
Source: PLoS One. 2017 Feb 3;12(2):e0171609. doi: 10.1371/journal.pone.0171609 (PMC5291516; doi:10.1371/journal.pone.0171609)
Supplement: S1 Appendix — (PDF) [file pone.0171609.s001.pdf]

Supporting Information

Human Mobility in Large Cities as a Proxy for Crime

Carlos Caminha[1],Vasco Furtado[1], Tarcisio H. C. Pequeno[1], Caio Ponte[1], Hygor P. M. Melo[2,3], Erneson A. Oliveira[1,3], José S. Andrade Jr.[3]

1 Programa de Pós Graduação em Informática Aplicada, Universidade de Fortaleza, Fortaleza, Ceará, Brasil

2 Departamento de Ensino, Instituto Federal de Educação, Ciência e Tecnologia do Ceará, Crateús, Ceará, Brasil

3 Departamento de Física, Universidade Federal do Ceará, Fortaleza, Ceará, Brasil

The urban mobility data processing

The urban mobility system of a large city is composed of several interconnected networks, such as subway, bus, bicycle, taxicab, and private vehicle networks. Buses are the main means of transportation for the most inhabitants in the city of Fortaleza, being used by about 700,000 people daily and we are able to reconstruct ~ 300k trajectories. According to IBGE (`http://www.cidades.ibge.gov.br/v3/cidades/municipio/2304400`), Fortaleza has ~ 556k cars and ~ 265k motorcycles. Supposing the upper bound for the daily commuting travels by cars and motorcycles (~ 821k), the total commuting travels in Fortaleza should be ~ 1.5M. Therefore, the bus system represents at least ~ 46% of the urban mobility within Fortaleza and we know the route of at least ~ 19% of the total commuting travels. Taking this fact into account, we assumed that the urban mobility within the city can be represented by the use of the bus system. Thus, the trajectories of bus users will be used to infer the floating population at the different points of the city.

In order to understand the people flow throughout the city, we used four spatio-temporal datasets related to Fortaleza's bus network, which are: bus stops; bus lines; GPS tracking of vehicles; and ticket card validation. All datasets refer to a normal business day, in fact, the March 11th, 2015 - a Wednesday. In total, Fortaleza has 4,783 bus stops served by 2,034 buses along 359 different routes. The integrated transportation model adopted by Fortaleza City Hall, called *Bilhete Único*, allows the registred users to make a bus transfer anywhere in the city, as long as it is within two hours since the last validation of their ticket card. The validation process is understood as the act of the user swiping his/her ticket card at the turnstile on the bus or at the bus terminal. Usually, such procedure happens at the beginning of the trip, since the turnstile is close to the bus entrance in Fortaleza. In this context, we are able to define the Origin-Destination Matrix (ODM) for Fortaleza's bus network through the following hypoteses [1]: We can assume that an user's

origin point could be represented by the earliest of all first daily ticket validations in the interval of two weeks before March 11th, as well as an user's destination point could also be represented by the earliest of all last daily ticket validations in the interval of one month before March 11th, bearing in mind that an user could have different destination points along the week, *i.e.* we have to analyze Mondays with Mondays, Tuesdays with Tuesdays, and so on. Hence, we estimated the origin-destination pair for about 40% of the bus users representing the overall behavior of the urban mobility in Fortaleza.

Finally, we supposed that the trajectories of bus users are defined by the composition of routes of the buses took by them between their origin-destination pair [2]. In this context, we describe the trajectories of bus users as a directed graph $G(V, E)$, where V and E are the set of vertices v and edges e, respectively. An edge e between the vertices v_i and v_j is defined by the ordered pair (v_i, v_j). In our approach, the vertices represent bus stops and the edges represent the demand of bus users between two consecutive bus stops. For each vertice v_i, we defined a weighted function $d(v_i)$ as the sum of the users passing by v_i. Thus, we calculated the floating population as the sum of all $d(v_i)$ within each census tracts.

Lack of correlation between POP × PC and FLO × DP.

In the main text, we can see the emergence of allometric relationships between POP × DP and FLO × PC in CCA clusters (Fig. 4). However, the same relationship between POP × PC and FLO × DP is not observed, as illustrated in S1 Fig. 1. The standard errors of β, represented by the shadows in the S1 Fig. 1, are greater than those found it in Fig. 4 showing the lack of correlation in these cases.

Additional information about the choosing of ℓ and D^*

In the main text, we observed the emergence of a trend toward an isometric relation between the populations (POP and FLO) of the CCA clusters and their respective areas (ARE). The S1 Fig 2 shows the closest relations to an isometric behavior for both cases. The CCA parameters used to define the clusters were $D^* = 6000$ and $\ell = 270$ for the POP case, illustrated in S1 Fig 2a, and $D^* = 2000$ and $\ell = 320$ for the FLO case in S1 Fig 2b.

Another approach to define $D*$ is by maximization of R^2. Unfortunately, R^2 showed not to be a

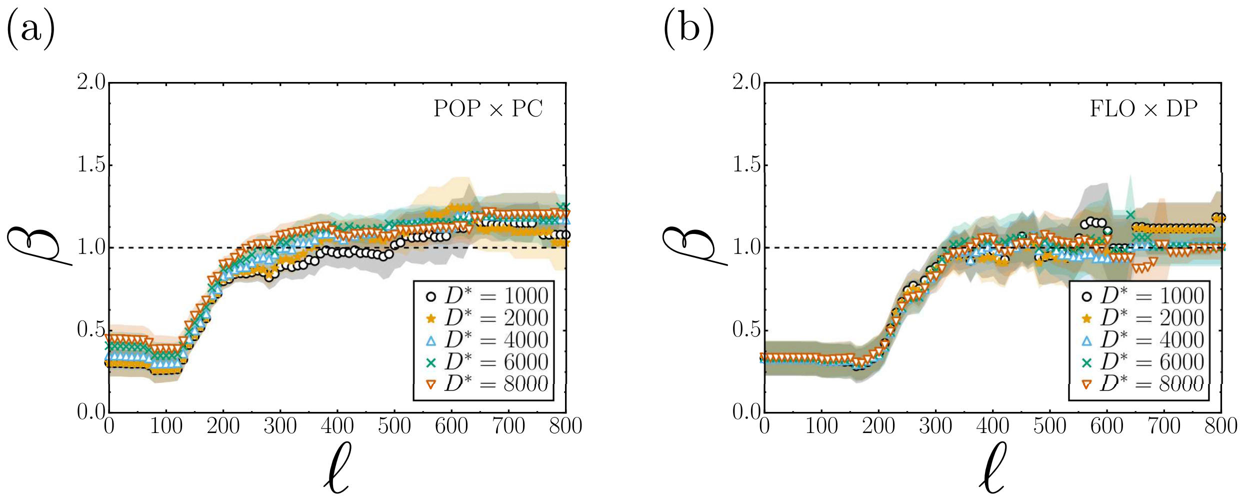

FIG. 1: **Behavior of the exponent β by varying the parameters of the City Clustering Algorithm (CCA), ℓ and D^*.** (a) The variation of β for the case between the resident population (POP) and the property crimes (PC) complaints. (b) The variation of β for the case between the floating population (FLO) and disturbing the peace (DP).

good indicator, it is quite constant when we change ℓ or D^*, as we can see in the S1 Fig 3. Another problem to use R^2 as an indicator arises when we note that an increasing in D^* can lead to an artificial increase on R^2 thanks to a sharp decrease on the number of cluster, leading to a fitting with only two or three points. In our approach, we seek to use the lowest value of D^* in order to have a large representation of the population on the scaling.

Data summary

Title	Size	Type	Data Availability
Bus Stops	122k	CSV	http://dados.fortaleza.ce.gov.br/catalogo/dataset/dados-de-onibus-11-03-2015
Bus GPS	236,7M	CSV	http://dados.fortaleza.ce.gov.br/catalogo/dataset/dados-de-onibus-11-03-2015
Smart Card Validation	77,8M	CSV	http://dados.fortaleza.ce.gov.br/catalogo/dataset/dados-de-onibus-11-03-2015
Crimes: PC and DP	6,3M	CSV	http://dados.fortaleza.ce.gov.br/dataset/8e995f96-423c-41f3-ba33-9ffe94aec2a8/resource/de4e876a-ee24-4d6e-9722-db9dc454bbe6/download/policecalls
Census Tract Boundaries	7,9M	KMZ	http://www.ibge.gov.br/english/
Resident Population	7,8M	CSV	http://www.ibge.gov.br/english/

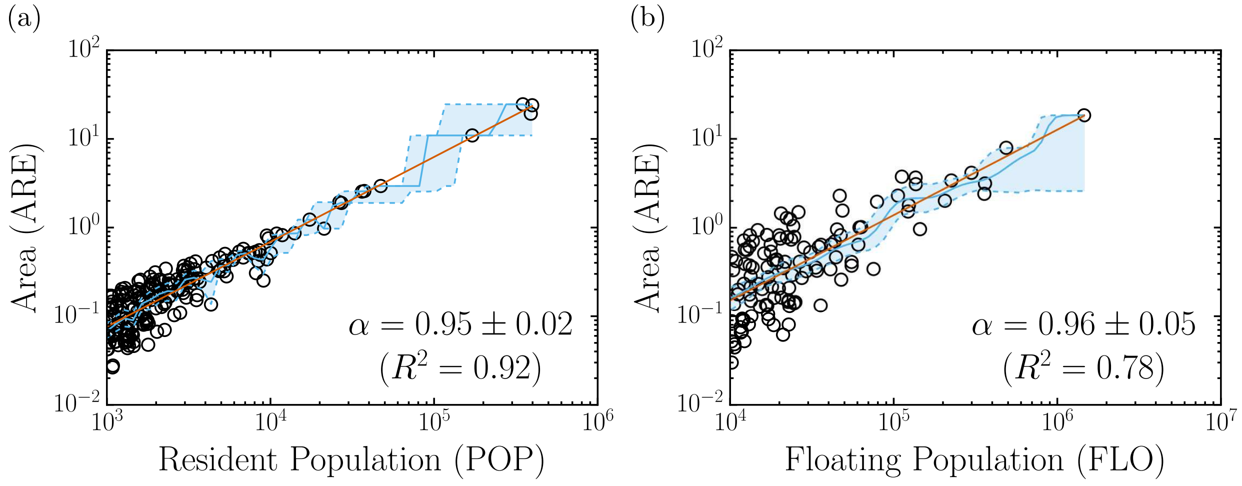

FIG. 2: **The isometric relation between resident (POP) and floating population (FLO) with area in square kilometers** (km^2)**.** (a) The correlation between POP and the area of the clusters (ARE) calculated using City Clustering Algorithm (CCA) where $D^* = 6000$ residents per km^2 and $\ell = 250$ meters (m). (b) The correlation between FLO and ARE calculated using the CCA for $D^* = 2000$ floating people per km^2 and $\ell = 320$ m. The red line shows the Ordinary Least Square (OLS) regression applied to the logarithm of the data [3, 4], and the blue continuous line indicates the Nadaraya-Watson kernel regression [5, 6]. Finally, the blue dashed lines delimit the 95% confidence interval estimated by 500 random bootstrapping samples with replacement [7, 8]. The R^2 is defined as the determination coefficient [3, 4].

---

[1] Caminha C, Furtado V, Pinheiro V, Ponte C. Micro-interventions in urban transportation from pattern discovery on the flow of passengers and on the bus network. In: Smart Cities Conference (ISC2), 2016 IEEE International. IEEE; 2016. p. 1–6.

[2] Caminha C, Furtado V, Pinheiro V, Ponte C. Mining the Networks of Supply and Demand of Public Transport for Overload and Waste of Resources. arXiv preprint arXiv:160603737. 2016;.

[3] Rawlings JO, Pantula SG, Dickey DA. Applied regression analysis: a research tool. Springer Science & Business Media; 2001.

[4] Montgomery DC, Peck EA, Vining GG. Introduction to linear regression analysis. John Wiley & Sons; 2015.

[5] Nadaraya EA. On estimating regression. Theory of Probability & Its Applications. 1964;9(1):141–142.

[6] Watson GS. Smooth Regression Analysis. Sankhyā: The Indian Journal of Statistics, Series A.

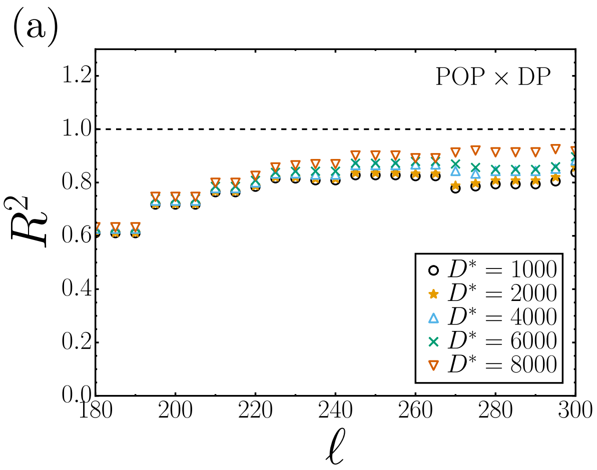

FIG. 3: **Behavior of R^2 by varying the parameters of the City Clustering Algorithm (CCA), ℓ and D^*.** (a) The variation of R^2 in correlation between the resident population (POP) and the disturbing the peace (DP) complaints is illustrated; (b) The variation is illustrated for correlations between the floating population (FLO) and the property crimes (PC).

1964;26(4):359–372.

[7] Racine J, Li Q. Nonparametric estimation of regression functions with both categorical and continuous data. Journal of Econometrics. 2004;119(1):99–130.

[8] Li Q, Racine J. Cross-validated local linear nonparametric regression. Statistica Sinica. 2004;14(2):485–512.
